# 288. Clinical Variables Associated with COVID-19 Mortality and ICU Admission in a Public Safety-net Hospital in Chicago

**DOI:** 10.1093/ofid/ofab466.490

**Published:** 2021-12-04

**Authors:** Ruben A Hernandez-Acosta, Juan Sarmiento, Palak Patel, Michael Hoffman, Katayoun Rezai

**Affiliations:** 1 John H. Stroger Jr. Hospital of Cook County / Cook County Health, Chicago, IL; 2 John H. Stroger Jr. Hospital of Cook County, Chicago, IL

## Abstract

**Background:**

The COVID-19 pandemic has disproportionately impacted minorities in the United States. John H. Stroger Jr. Hospital (JSH) is a tertiary care hospital within the safety-net system for Cook County in Chicago, Illinois. In this study we report demographics, clinical characteristics and outcomes of patients admitted with COVID-19 in our hospital during the spring surge of 2020.

**Methods:**

A retrospective study was done including patients > 18 years of age admitted to JSH with positive PCR for SARS-CoV2 from March 18 to May 30th, 2020. Outcomes, clinical and demographic characteristics were extracted from the electronic medical record. Moderate and severe disease were defined as radiographic evidence of pulmonary infiltrates and SpO2 > 94% on room air or SpO2< 94% on room air, respectively. Bivariate analysis and logistic regression were performed to assess for risk factors for admission to the intensive care unit and mortality.

**Results:**

625 patients were included, 424 (68%) were male. Median age was 44 years (44,63). 364 (58%) were Hispanic and 222 (36%) non-Hispanic Blacks. 113 (18%) of patients presented with mild disease, 204 (33%) with moderate disease, 298 (48%) with severe disease. 73 patients (12%) died. 153 (24%) required ICU admission, 84 (13%) required intubation [Table 1]. In bivariate analysis, increasing age and diabetes (DM) were associated with increased mortality and ICU admission (p=0.001, Tables 2 and 3). Race/ethnicity was not associated with increased mortality or ICU admission. In the multivariate analysis, elevated glucose on admission regardless of DM and CKD were associated with mortality (p < 0.001).

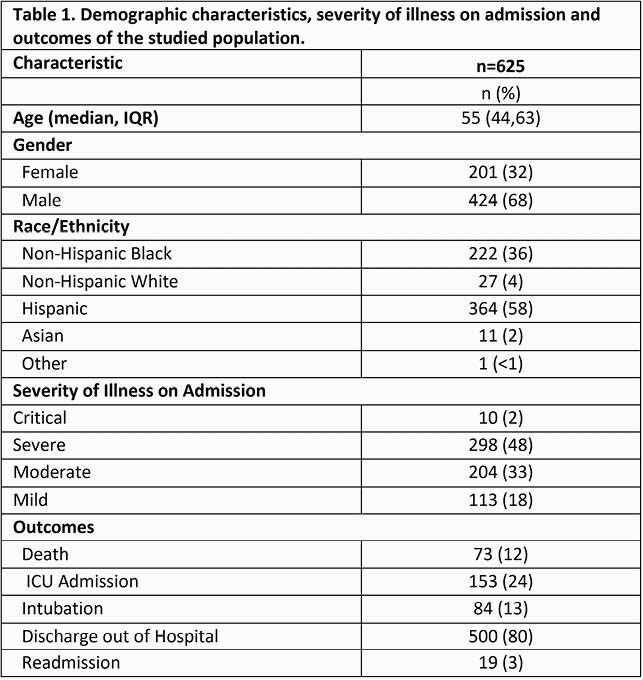

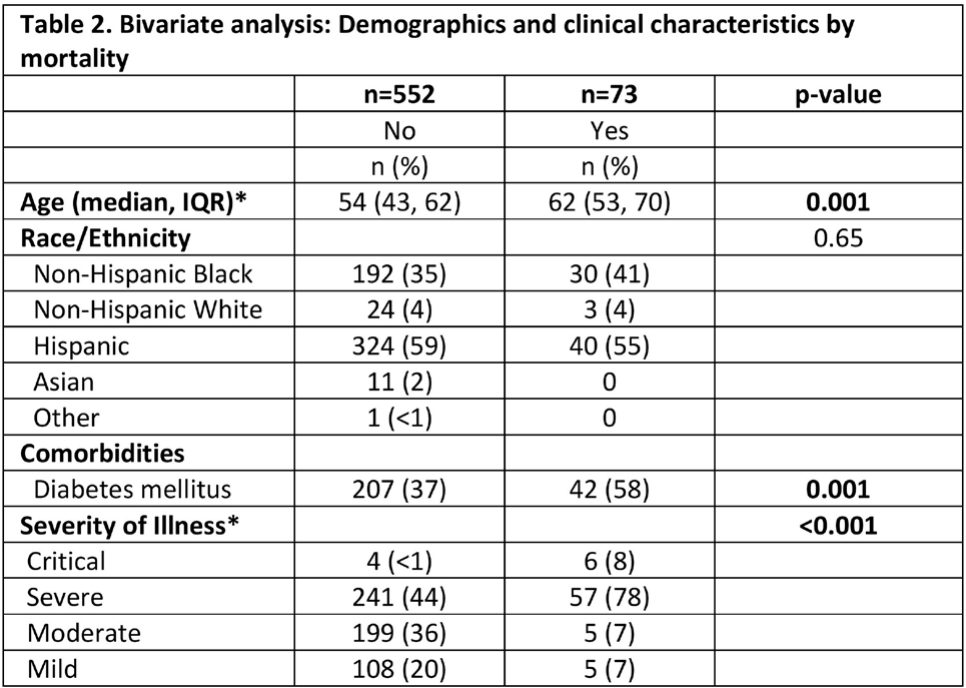

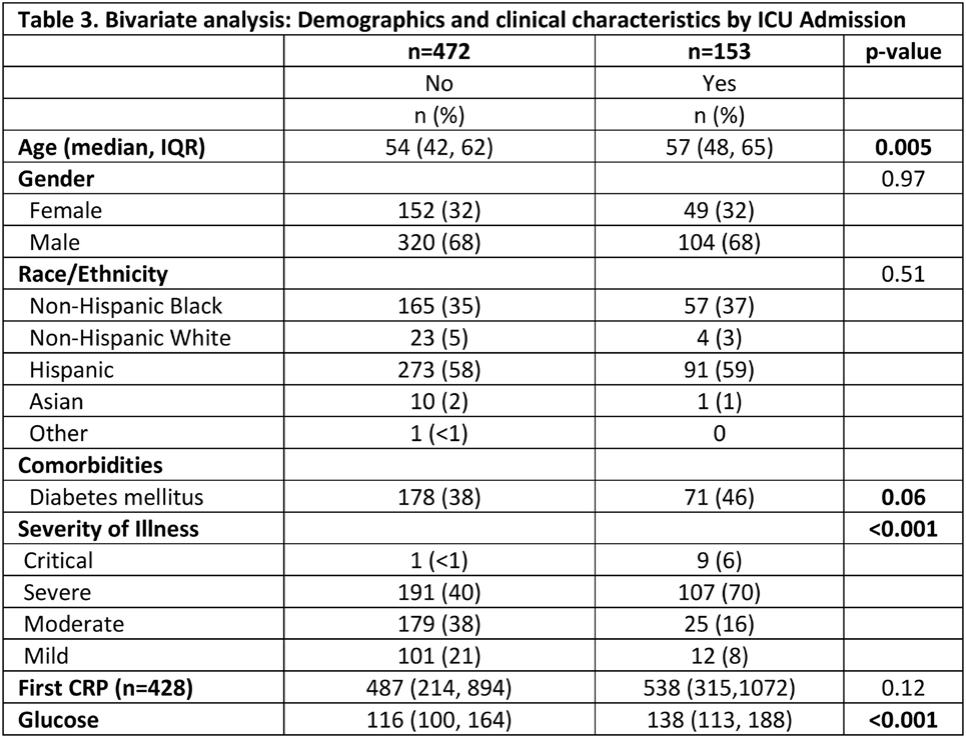

**Conclusion:**

JSH is a safety net hospital that provides care for the most vulnerable population of Chicago. The proportion of Hispanic patients increased in the later weeks of the pandemic until they represented most of the inpatient population and presented with more severe disease (Figure 1). Although race was not associated with mortality or ICU admission, the high prevalence of chronic diseases such as hypertension and DM in our population may explain the higher rate of admissions. Strengthening of preventive medicine and social engagement with minorities must be a crucial effort to decrease the burden of COVID-19 in this population.

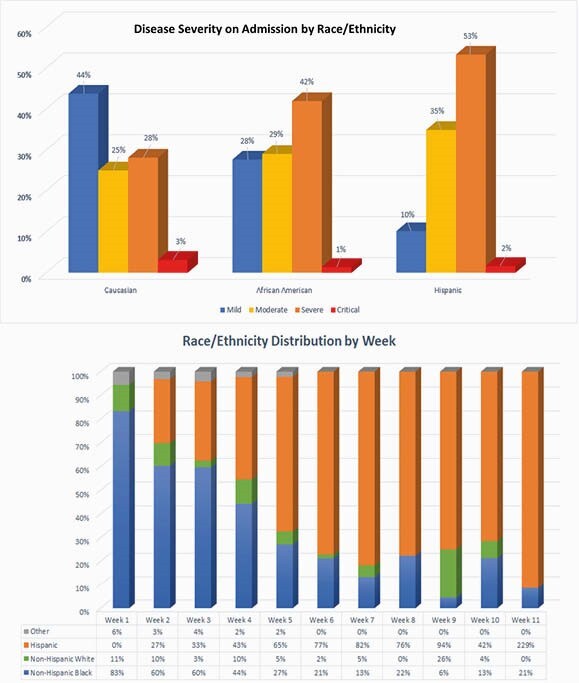

Graph showing disease severity on admission by Race/Ethnicity (upper). Notice the predominance of severe disease (orange) in Hispanic patients. Graph showing Race/Ethnicity Distribution by Week (lower). Notice the gradual increase and predominance of Hispanic patients (orange) in the later weeks of the study period compared to Black (blue) and White (green) patients.

**Disclosures:**

**All Authors**: No reported disclosures

